# Association of a Disrupted Dipping Pattern of Blood Pressure with Progression of Renal Injury during the Development of Salt-Dependent Hypertension in Rats

**DOI:** 10.3390/ijms21062248

**Published:** 2020-03-24

**Authors:** Abu Sufiun, Asadur Rahman, Kazi Rafiq, Yoshihide Fujisawa, Daisuke Nakano, Hideki Kobara, Tsutomu Masaki, Akira Nishiyama

**Affiliations:** 1 Department of Pharmacology, Faculty of Medicine, Kagawa University, Kagawa 761-0793, Japan; sufian04@yahoo.com (A.S.); rahmanma@med.kagawa-u.ac.jp (A.R.); krafiq73@yahoo.com (K.R.); dnakano@med.kagawa-u.ac.jp (D.N.); 2 Life Science Research Center, Faculty of Medicine, Kagawa University, Kagawa 761-0793, Japan; recfuji@kms.ac.jp; 3 Department of Gastroenterology, Faculty of Medicine, Kagawa University, Kagawa 761-0793, Japan; kobara@med.kagawa-u.ac.jp (H.K.); tmasaki@med.kagawa-u.ac.jp (T.M.)

**Keywords:** blood pressure (BP), dipping pattern of BP, Dahl salt-sensitive (DSS) rats, proteinuria, renal injury

## Abstract

The aim of the present study is to investigate whether a disruption of the dipping pattern of blood pressure (BP) is associated with the progression of renal injury in Dahl salt-sensitive (DSS) hypertensive rats. Seven-week-old DSS rats were fed a high salt diet (HSD; 8% NaCl) for 10 weeks, followed by a transition to a normal salt diet (NSD; 0.3% NaCl) for 4 weeks. At baseline, NSD-fed DSS rats showed a dipper-type circadian rhythm of BP. By contrast, HSD for 5 days caused a significant increase in the difference between the active and inactive periods of BP with an extreme dipper type of BP, while proteinuria and renal tissue injury were not observed. Interestingly, HSD feeding for 10 weeks developed hypertension with a non-dipper pattern of BP, which was associated with obvious proteinuria and renal tissue injury. Four weeks after switching to an NSD, BP and proteinuria were significantly decreased, and the BP circadian rhythm returned to the normal dipper pattern. These data suggest that the non-dipper pattern of BP is associated with the progression of renal injury during the development of salt-dependent hypertension.

## 1. Introduction

In healthy subjects, blood pressure (BP) follows a diurnal variation, with a physiological dipping (>10%) during the nighttime (inactive) compared with the daytime (active) [1]. Conversely, in hypertensive patients with chronic kidney disease (CKD), BP fails to dip during the inactive period, resulting in an atypical circadian rhythm of BP termed the non-dipper pattern of BP [2]. Importantly, the prevalence of the case of non-dipper BP in subjects with hypertension is approximately 30% [3]. Moreover, the non-dipper pattern of BP is closely associated with a greater risk of cardiovascular complications such as left ventricular hypertrophy, cerebrovascular disease, microalbuminuria, and end-stage renal diseases [4,5,6].

There is a close relationship between salt-sensitive hypertension and the non-dipper pattern of BP [7,8]. Irrespective of the mechanism [4,9], the inactive-period dip in BP is diminished in patients with salt-dependent hypertension. Interestingly, a week of sodium restriction [10] or administration of diuretics [11] normalized the dipping pattern of BP in essential hypertensive patients who exhibited a non-dipper profile of BP. Importantly, salt-independent hypertensive patients showed a normal inactive-period dip in BP, which was not modified by the low-salt diet or diuretics [10]. Furthermore, the circadian rhythm of urinary sodium excretion is strongly related to the diurnal variation of BP. In particular, the night/day ratio of sodium excretion was lower in dippers, while the ratio was greater in non-dippers [12]. In this regard, Bankir et al. [1] reported that the capacity to excrete sodium during the daytime is a significant determinant of nocturnal BP and dipping. Collectively, these data suggest that alterations in the normal nocturnal or inactive-period dip in BP are associated with salt sensitivity of BP, as well as high sodium intake.

A growing body of clinical evidence suggests that patients with CKD often develop salt-dependent hypertension with a non-dipper type of BP [2,13]. We recently reported that normal salt diet (NSD; 0.3% NaCl)-fed Dahl salt-sensitive (DSS) rats did not develop hypertension and exhibited a normal dipping pattern of BP (active period > inactive period) [14]. By contrast, high salt diet (HSD; 8% NaCl) feeding for 1 week further increased the active period BP, as well as the difference in the active and inactive periods, suggesting an extreme dipper pattern of BP. Furthermore, 1 week after switching to an NSD, the active period BP decreased and exhibited a normal dipping pattern. These data suggest that short-term treatment with an HSD increases BP with an extreme dipper pattern, rather than a non-dipper pattern, during the early phase of the development of salt-dependent hypertension in DSS rats. Therefore, we hypothesize that the occurrence of a non-dipper pattern of BP in chronic cases is associated with deterioration of renal function in DSS rats. To test this hypothesis, we continuously monitored BP during the progression of proteinuria as well as renal injury in DSS hypertensive rats.

## 2. Results

### 2.1. Changes in Body Weight, BP, and Heart Rate

**Figure 1:**
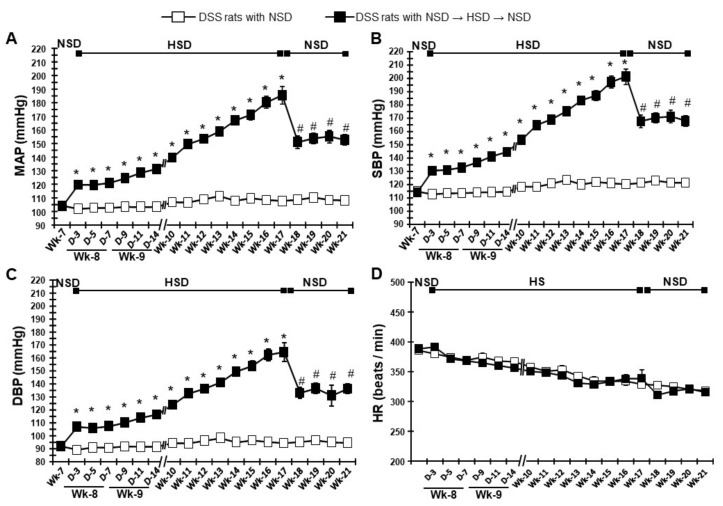
Time-dependent changes of blood pressure and heart rate. Averaged 20-h (**A**) mean arterial pressure (MAP), (**B**) systolic blood pressure (SBP), (**C**) diastolic blood pressure (DBP), and (**D**) heart rate (HR) during feeding normal salt diet (NSD, 0.3% NaCl, week 7), high salt diet (HSD, 8% NaCl, weeks 8–17), and again after switching to NSD (week 18–21) in Dahl salt-sensitive (DSS) rats. * *p* < 0.05 vs. DSS rats with NSD; ^#^ *p* < 0.05, DSS rats with NSD→HSD→NSD (week 17) vs. DSS rats with NSD→HSD→NSD (week 18–21).

As shown in Figure S1, body weight gain was similar in both the HSD- and NSD-fed DSS rats during the experimental period. However, HSD feeding for 10 weeks increased the mean arterial pressure (MAP; 185 ± 6 mmHg at 17 weeks of age) in a time-dependent manner, while continuous NSD feeding did not alter MAP (107 ± 2 mmHg at 17 weeks of age) in DSS rats (Figure 1A). Following 10 weeks of HSD, it was switched to NSD, and MAP was rapidly decreased (152 ± 3 mmHg at 21 weeks of age). However, MAP remained significantly higher than that for continuously NSD-fed DSS rats (*p* < 0.05). Similarly, HSD feeding for 10 weeks significantly increased systolic BP (SBP; 201 ± 5 mmHg) and diastolic BP (164 ± 7 mmHg) in DSS rats (Figure 1B,C). At 4 weeks after switching to the NSD, both SBP (167 ± 4 mmHg) and diastolic BP (136 ± 3 mmHg) followed the same trend as for MAP. HR gradually decreased in both the HSD- and NSD-fed DSS rats. By contrast, switching from the HSD to the NSD at 17 weeks of age caused a sudden decline in HR, and after 4 weeks of NSD, HR was similar to that in the continuously fed-NSD animals (Figure 1D).

### 2.2. Changes in the Dipping Pattern of BP

#### 2.2.1. Dipping Pattern of BP During Feeding NSD 

**Figure 2:**
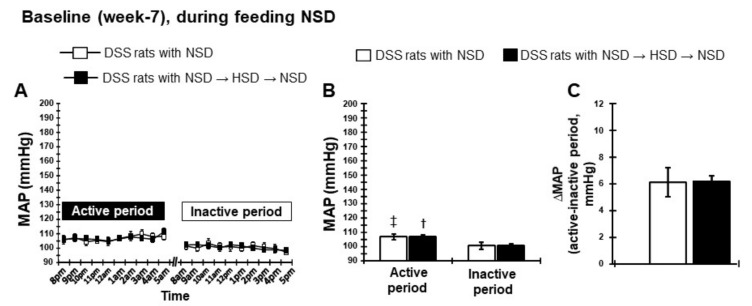
Circadian rhythm of MAP at baseline during feeding normal salt diet (NSD, 0.3% NaCl diet at week 7). (**A**) Hourly MAP with NSD, (**B**) averaged 10-h MAP in active and inactive periods, and (**C**) the difference of 10-h MAP between active and inactive periods. ^†^ *p* < 0.05, DSS rats with NSD→HSD→NSD (inactive period) vs. DSS rats with NSD→HSD→NSD (active period); ^‡^ *p* < 0.05; DSS rats with NSD (inactive period) vs. DSS rats with NSD (active period).

At baseline (7 weeks of age), NSD-fed DSS rats exhibited higher MAP in the active (dark) period compared with the inactive (light) period (Figure 2A). Moreover, the averaged 10-h MAP during the active period was significantly higher than that in the inactive period (Figure 2B,C), indicating that the normotensive DSS rats showed a dipper-pattern of BP.

#### 2.2.2. Dipping Pattern of BP During Feeding HSD 

**Figure 3:**
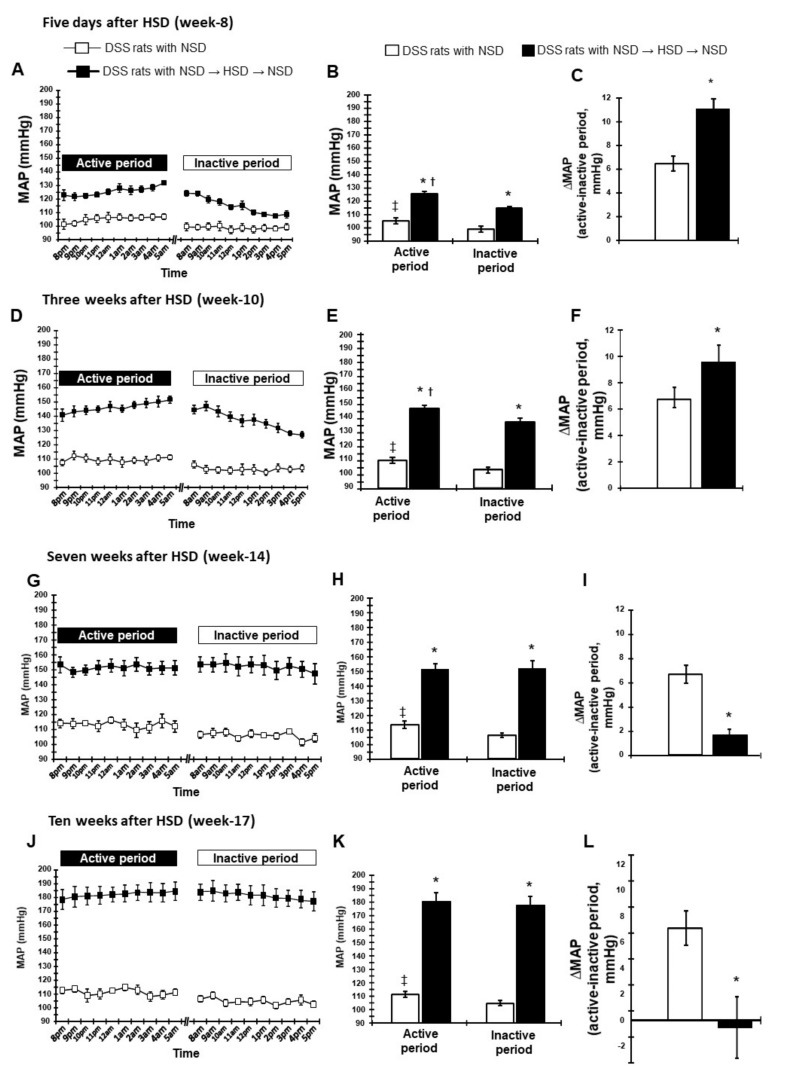
Circadian rhythm of MAP during feeding HSD (week 8–17). (**A**) Hourly MAP, (**B**) averaged 10-h MAP in active and inactive periods, and (**C**) the difference of 10-h MAP between active and inactive periods after 5 days of HSD. (**D**) Hourly MAP, (**E**) averaged 10-h MAP in active and inactive periods, and (**F**) the difference of 10-h MAP between active and inactive periods after 3 weeks of HSD. (**G**) Hourly MAP, (**H**) averaged 10-h MAP in active and inactive periods, and (**I**) the difference of 10-h MAP between active and inactive periods after 7 weeks of HSD. (**J**) Hourly MAP, (**K**) averaged 10-h MAP in active and inactive periods, and (**L**) the difference of 10-h MAP between active and inactive periods after 10 weeks of HSD in DSS rats. * *p* < 0.05 vs. DSS rats with NSD; ^†^ *p* < 0.05, DSS rats with NSD→HSD→NSD (inactive period) vs. DSS rats with NSD→HSD→NSD (active period); ^‡^ *p* < 0.05, DSS rats with NSD (inactive period) vs. DSS rats with NSD (active period).

Five days feeding of the HSD in DSS rats caused a further increase in the active period MAP (126 ± 2 mmHg) compared with baseline (Figure 3A). Although inactive period MAP increased (115 ± 2 mmHg) at the same time in HSD-fed DSS rats (Figure 3B), the difference between the active and inactive period MAP was further increased compared with the NSD-fed DSS rats (11.1 ± 0.9 vs. 6.5 ± 0.6 mmHg, respectively; *p* < 0.05; Figure 3C), suggesting an extreme dipper-type of BP in HSD-fed DSS rats. Although the difference between the active and inactive period MAP tended to decrease after 3 weeks of HSD compared with the NSD-fed DSS rats, the extreme dipping pattern of BP was maintained at this time point (Figure 3D−F).

Interestingly, the dipping pattern of BP tended to change to the non-dipping pattern after HSD feeding for 7 weeks in DSS rats (Figure 3G−I). At this time point, there were no differences in MAP between the active and inactive periods (180 ± 6 vs. 178 ± 6 mmHg, respectively; Figure 3H). Importantly, the difference in MAP between the active and inactive periods in HSD-fed DSS rats was significantly lower compared with that in NSD-fed rats (1.8 ± 0.3 vs. 6.7 ± 0.7 mmHg, respectively; *p* < 0.05, Figure 3I). Ten weeks after feeding the HSD, MAP further increased in both the active and inactive periods, although the difference between the active and inactive periods decreased compared with the 7-week HSD-feeding time point (Figure 3J−L). This difference in MAP was also significantly lower in HSD-fed rats compared with NSD rats. These data suggest that HSD feeding for a longer period results in a non-dipping pattern of BP in DSS rats.

#### 2.2.3. Dipping Pattern of BP After Transition of HSD to LSD

**Figure 4:**
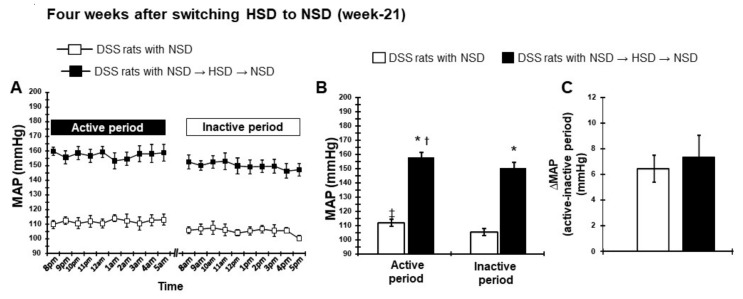
Circadian rhythm of MAP after switching to NSD (week 18–21). (**A**) Hourly MAP, (**B**) averaged 10-h MAP in active and inactive periods, and (**C**) the difference of 10-h MAP between active and inactive periods at 4 weeks after switching HSD to NSD. * *p* < 0.05 vs. DSS rats with NSD; ^†^ *p* < 0.05, DSS rats with NSD→HSD→NSD (inactive period) vs. DSS rats with NSD→HSD→NSD (active period); ^‡^ *p* < 0.05, DSS rats with NSD (inactive period) vs. DSS rats with NSD (active period).

Following the transition of HSD to NSD, MAP declined dramatically within 1 week and then stabilized for the remainder of the experiment for 4 weeks (Figure 4A). Interestingly, the difference in the active period (157 ± 4 mmHg) and inactive period (150 ± 4 mmHg) MAP was increased by feeding NSD for 4 weeks following a transition from HSD (Figure 4B). Moreover, the difference in the active and inactive period MAP was similar between the salt transition rats fed the NSD for 4 weeks and those continuously fed the NSD (Figure 4C), suggesting a normalization of the dipping pattern of BP. Similar changes in SBP were observed during the experimental period (Figures S2−S7).

### 2.3. Changes in Proteinuria and its Association with the Dipping Pattern of BP

**Figure 5:**
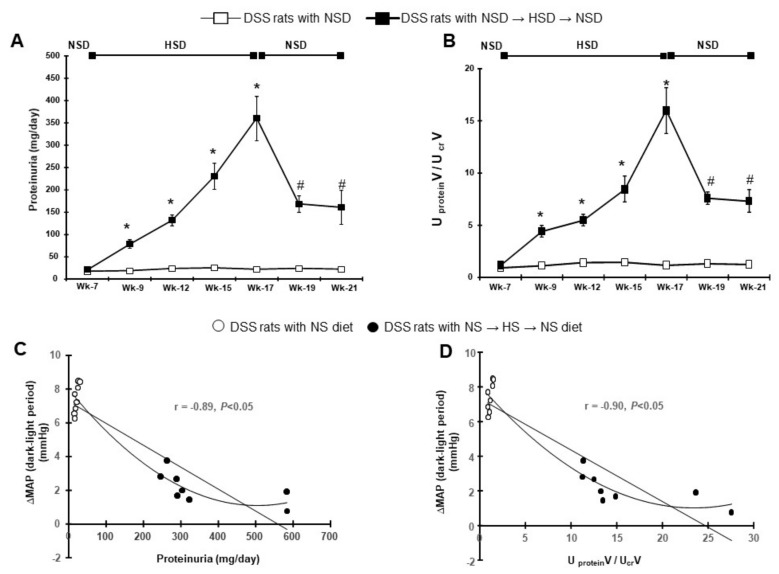
Effects of HSD on urinary protein excretion. (**A**) The 24-h urinary protein excretion, and (**B**) the urinary protein and creatinine (UproteinV/Ucr.V) ratio. (**C**) Correlation between differences in active and inactive period MAP and level of urinary protein excretion. (**D**) Correlation between differences in active and inactive period MAP and urinary protein–creatinine ratio. * *p* < 0.05 vs. DSS rats with NSD; ^#^ *p* < 0.05, DSS rats with NSD→HSD→NSD (week 17) vs. DSS rats with NSD→HSD→NSD (week 18–21).

The level of urinary protein excretion increased in HSD-fed DSS rats compared with those fed with the NSD during salt loading for 10 weeks (Figure 5A). DSS rats with HSD showed marked proteinuria (360 ± 50 mg/day) compared with age-matched NSD-fed rats (21 ± 2 mg/day) at 17 weeks of age. The transition of salt loading from the HSD to the NSD caused a dramatic reduction in the level of proteinuria at 19 weeks of age. Following 4 weeks of NSD feeding, urinary protein excretion (160 ± 38 mg/day) was maintained at levels similar to those at 19 weeks of age. Nevertheless, urinary protein excretion levels remained higher than those of age-matched continuous NSD-fed DSS rats (22 ± 2 mg/day). The temporal ratio of urinary protein excretion and urinary Cr is shown in Figure 5B. A gradual increase in urinary protein excretion was observed in DSS rats during the 10 weeks of HSD. The urinary protein and Cr ratio in HSD-fed rats (16.0 ± 2.0 g/g) followed the same trend as for age-matched NSD-fed DSS rats (1.2 ± 0.1 g/g). After transition to NSD, urinary protein excretion, and the urinary protein and Cr ratio, dropped within 2 weeks, and then stabilized for the remainder of the experiments.

The relationship between differences in the active period and inactive period MAP and urinary protein excretion is shown in Figure 5C,D. At 10 weeks after feeding the HSD, the difference in MAP between the active period and the inactive period was negatively correlated with urinary protein excretion at 17 weeks of age (r = −0.89, *p* < 0.05; Figure 5C). Importantly, there was also a negative correlation of the difference in MAP (active and inactive periods) with the urinary protein and Cr ratio at 17 weeks of age (r = −0.90, *p* < 0.05; Figure 5D). Similarly, SBP was negatively correlated with urinary protein excretion (r = −0.88, *p* < 0.05; Figure S8A) and the urinary protein and Cr ratio (r = −0.85, *p* < 0.05; Figure S8B) at this age.

### 2.4. Renal Tissue Injury and Dipping Pattern of BP in Salt Loaded-DSS Rats

**Figure 6:**
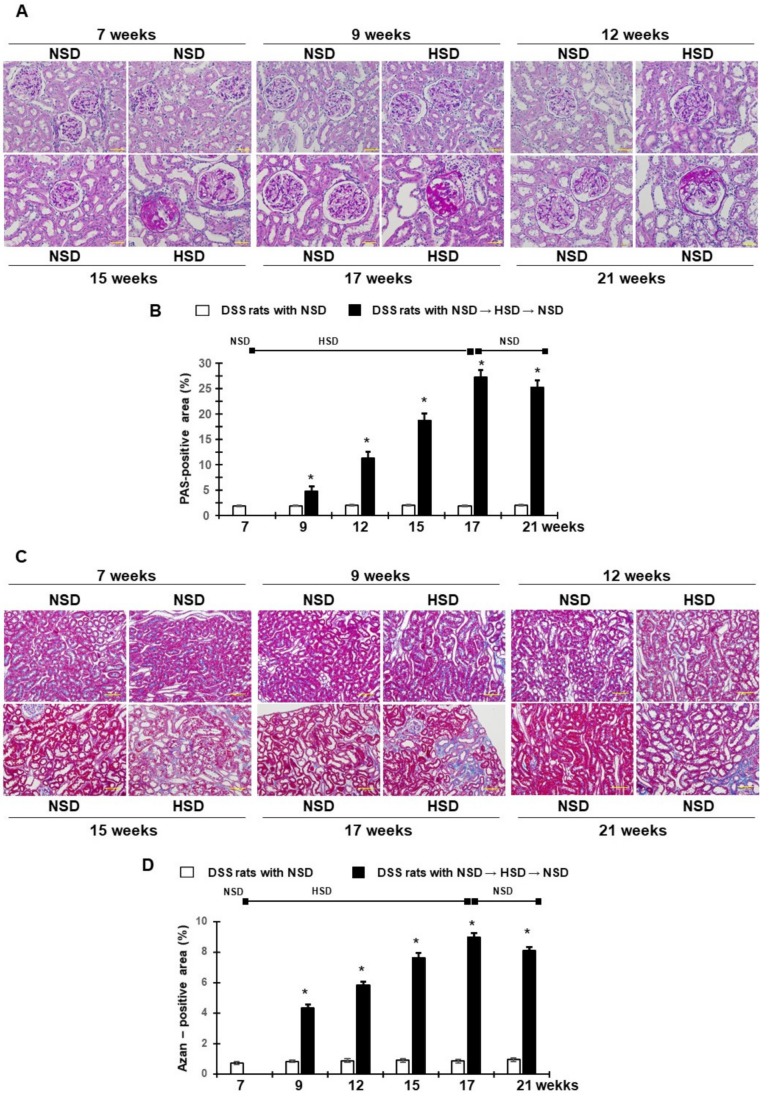
Effects of HSD on renal glomerular and tubulointerstitial injury. (**A**) Representative’s images of PAS-stained renal sections (magnification, ×200; scale bar 50 μm). (**B**) The PAS-positive area within the glomerular area. (**C**) Representative micrographs of Azan-stained renal sections (magnification, ×100, scale bar 100 μm). (**D**) Quantitative analysis of Azan-positive area. * *p* < 0.05 vs. DSS rats with NSD.

Photomicrographs of glomeruli stained with PAS and quantitative analyses of the glomerular-positive area are shown in Figure 6A,B, respectively. NSD-fed DSS rats showed normal glomeruli or very slight glomerular damage during the observation period. By contrast, HSD feeding for 10 weeks resulted in glomerulosclerosis in DSS rats, as assessed by an increase in the glomerular PAS-positive area. Ten weeks of HSD feeding and subsequent transition to NSD did not affect the HSD-induced increase in glomerulosclerosis (Figure 6A,B).

Photomicrographs of the tubulointerstitium with Azan staining and quantitative analyses of the positive area for tubulointerstitial fibrosis are shown in Figure 6C,D, respectively. Renal tubulointerstitial fibrosis showed a time-dependent progression during the HSD regimen in DSS rats. After 10 weeks of HSD feeding, marked tubulointerstitial fibrosis was observed in DSS rats compared with NSD rats. After switching to the NSD following the HSD regimen, there was no further change in tubulointerstitial fibrosis until the end of the experiments (Figure 6C,D). Interestingly, the difference in the active and inactive period SBP was negatively correlated with the PAS-positive area (r = −0.91, *p* < 0.05; Figure S9A) and the Azan positive area (r = −0.90, *p* < 0.05; Figure S9B).

### 2.5. Urinary Excretion of Sodium, Plasma Cr, and BUN

As expected, DSS rats on the HSD showed a higher 24-h urine volume (Figure S10) and urinary sodium excretion (Table 1) compared with the NSD-fed rats. After switching to the NSD, the 

Sodium excretion rate was significantly decreased (*p* < 0.05). Plasma levels of creatinine and blood urea nitrogen (BUN) were not changed during the observation period (Table 1).

## 3. Discussion

Salt-sensitive hypertension is characterized by an increase in BP in response to increased dietary salt intake and is associated with an enhanced risk of renal and cardiovascular morbidity [2]. In the present study, salt loading in DSS rats caused an increase in BP both in active and inactive periods. During the early phase of hypertension, HSD-fed DSS rats showed an extreme dipper pattern of BP. These data are consistent with that observed in a previous study in DSS rats [14]. However, continuous feeding of HSD led to the development of a non-dipper pattern of BP during the progression of renal injury, as evidenced by an increase in urinary protein excretion. These data indicate that a non-dipper pattern of BP is associated with deterioration of renal function during the development of salt-dependent hypertension.

Clinical evidence supports a close relationship between salt sensitivity of BP and the non-dipper pattern of BP [7,15]. We previously reported that 1 week of salt loading in DSS rats caused an extreme dipper pattern of BP [14]. We have also consistently shown that HSD-fed DSS rats exhibit an increase in active period BP, resulting in an increase in the differences between the active and inactive period BP compared with NSD-fed rats until 3 weeks of recovery. Strengthening these findings, our present data suggest that the extreme dipper pattern of BP is the earlier event in the course of dipping pattern changes in salt-sensitive hypertension. Interestingly, 7 weeks of salt loading in DSS rats diminished the differences in the active and inactive period BP compared with NSD-fed DSS rats, suggesting a non-dipper profile of BP. Subsequently, 10 weeks of salt loading caused a further increase in inactive-period BP compared with active-period BP and maintained the non-dipper pattern of BP. Within 1 week of transition to the NSD, the diurnal variation of BP returned to the dipper type, and this was maintained until the end of the experiment. A limitation of this study is that we evaluated the dipping pattern of BP and renal injury during the development of salt-sensitive hypertension in DSS rats upon salt loading without any therapeutic intervention.

Several renal and extra-renal mechanisms are believed to play a role in the non-dipper pattern of BP in salt-sensitive hypertension [16]. The diurnal variation of BP and night/day ratios of urinary sodium excretion have a strong positive relationship in patients on an HSD, but not in patients on an LSD, suggesting that sodium excretion is dependent on systemic BP in patients with a high salt intake (especially in non-dippers) [12]. Consistent with these clinical findings, we observed a dramatic increase in the 24-h urinary excretion of sodium after salt loading. Moreover, in patients with salt-sensitive hypertension and CKD, the diminished or altered sodium excretory capability determines the diurnal variation of BP [17]. After high salt loading, the defect in sodium excretory capacity becomes evident, resulting in elevated nighttime BP, which contributes to the non-dipper type of BP. This non-dipper pattern compensates for diminished natriuresis in patients during the daytime (active period) and enhances pressure natriuresis during the nighttime (inactive period) [2,18]. Moreover, restricted dietary-sodium intake is widely recommended for treating hypertension [19]. Previous clinical studies have shown that sodium restriction [10] and diuretics [11] can normalize the BP circadian rhythm from the non-dipper to the dipper type. In the present study, BP decreased after switching from the HSD to the NSD in DSS rats. Interestingly, NSD normalized the BP from non-dipper to dipper in DSS hypertensive rats.

Proteinuria is an important biomarker for the development of CKD [20,21]. Urinary albumin excretion is also significantly greater in non-dipper patients compared with the dipper type [22]. Consistent with previous reports [23,24], the present study showed that 10 weeks of high salt loading caused a gradual increase in proteinuria in DSS rats. Importantly, in the initial period of salt loading, when DSS rats exhibited an extreme dipper pattern of BP, urinary protein excretion was significantly different from that in NSD-fed DSS rats. There was also a time-dependent development of glomerulosclerosis during the course of high salt loading in DSS rats. Further, there was a significant negative correlation of differences in MAP (active and inactive period) with urinary protein excretion. Accumulating evidence suggests that in the sodium-sensitive type of essential hypertension, glomerular capillary pressure is elevated, and urinary albumin excretion is greater than that in the non-sodium-sensitive type [25,26]. Moreover, Fukuda et al. reported that the nocturnal dip in BP is lost in glomerulopathy, resulting in enhanced urinary protein excretion during the night (inactive period) [27]. These data suggest that urinary protein excretion plays an important role in the circadian rhythm of BP in DSS rats, which is consistent with the clinical notion that the rise in nocturnal BP with progressive loss of kidney function leads to proteinuria. Although the glomerular filtration rate (GFR) is considered an important determinant of CKD, a limitation of hypertensive animal studies is that an obvious reduction in GFR is not usually observed.

Tubulointerstitial fibrosis is suggested to be the most relevant renal histological parameter associated with nocturnal hypertension [8]. In the present study, salt-induced hypertension aggravated tubulointerstitial injury. After 10 weeks of HS loading, there was a significant negative correlation of differences in MAP (active and inactive) with tubulointerstitial fibrosis. These data suggest that the degree of renal function loss was closely correlated with the degree of non-dipping of BP, which is supported by previous reports [27,28]. Moreover, HS loading in DSS rats was reported to cause a gradual increase in the expression of α-SMA, TGF-β, and collagen-1 (fibrosis markers), desmin, nephrin, and podocin (glomerular podocyte injury markers), mononuclear cells, MCP-1 and PAI-1 (inflammatory cell markers), and 4-HNE (oxidative stress marker) in the kidney [23,29], which may be involved in the changes of the dipping pattern of BP.

A suppressed systemic renin-angiotensin system is commonly seen in subjects with salt-dependent hypertension [29]. Consistently, we have shown that HSD-fed DSS rats exhibit a marked decrease in plasma angiotensin II levels, accompanied by reduced plasma renin activity (PRA) [30,31]. Therefore, the reduced RAS activity may not contribute to the pathogenesis of the dipping pattern of BP in HSD-fed DSS rats.

In conclusion, the present study demonstrated that high salt loading initially caused an extreme dipper pattern of BP in DSS rats and changed to the non-dipper pattern of BP during the development of renal injury in the chronic course of salt loading. However, after cessation of HSD, hypertension and proteinuria were improved, but remained significantly higher compared with the NSD-fed animals. These data suggest that salt restriction is crucial for maintaining the normal dipping pattern of BP in subjects with salt-sensitive hypertension. These observations may help to develop therapeutic strategies to normalize the diurnal variation of BP in salt-dependent hypertension.

## 4. Materials and Methods

### 4.1. Experimental Animals

Experimental protocols (protocol no. 18627, date: 1 April 2015) were approved by the Animal Experimentation Ethics Committee at Kagawa University. All experimental procedures were performed according to the guidelines for care and use of animals established by Kagawa University. Male 4-week-old DSS rats (*n* = 64) were obtained from SLC Japan (Hamamatsu, Japan). Rats were housed in specific pathogen-free animal facilities under controlled temperature (24 ± 2 °C) and humidity (55% ± 5%) with a 12 h light–dark cycle. All animals were maintained on an NSD (0.3% NaCl) until 7 weeks of age and were divided into BP measurement with telemetry system, urinary protein and sodium excretion measurement, and renal histological analyses groups.

Group 1: BP measurement with a telemetry system.

To measure BP continuously in conscious animals, a telemetry system (Data Science International, Saint Paul, MN, USA) was used, as described previously [14,32]. Briefly, 6-week-old rats were anesthetized with isoflurane, and an abdominal incision was made for implantation of the radio-telemetry device. The system consists of a radiofrequency transmitter (TA11PA-C40), a receiver panel (RPC-1), an adaptor (R11CPA), and an ambient pressure monitor (APR-1; Data Science International). The telemetry transmitter catheter was positioned into the abdominal aorta and glued (3M vetbond; 3M Animal Care Products, Saint Paul, MN, USA) into position. The transmitter was secured to the abdominal wall with sutures. Data were collected and analyzed using Dataquest ART version 4.3 (Data Science International, New York, USA). At 1 week after surgery, baseline BP was measured continuously by the telemetry system at 7 weeks of age under the NSD (*n* = 18). DSS rats were then randomized into two groups. In one group (*n* = 8), animals were maintained uninterrupted on the NSD for 14 weeks. In the other group (*n* = 10), the NSD was switched to the HSD and continued for 10 weeks, and then switched again to the NSD for 4 weeks. In these animals, BP was measured continuously (5 min of automatic sampling per h) by the telemetry system every 1–4 weeks.

Group 2: Measurement of proteinuria and urinary sodium excretion with metabolic cages.

In a separate group of animals, we investigated the time-dependent changes in proteinuria and urinary sodium excretion in DSS rats. Seven-week-old DSS rats were treated with the NSD (*n* = 8) or the HSD (*n* = 8). DSS rats-fed the HSD were switched to the NSD and continued for 4 weeks, while other DSS rats received an uninterrupted NSD. After a 12-h acclimatization period in the metabolic cages, 24-h urine samples were collected at 7 weeks of age (at baseline, before initiating HSD), at 9, 12, 15, and 17 weeks of age (during HSD feeding), and at 21 weeks of age (4 weeks after switching to the NSD). Urinary protein and creatinine (Cr) levels were measured using commercial assay kits (Wako Pure Chemical Industries, Ltd. Osaka, Japan). Urinary sodium was measured by an automated analyzer machine (7020-Automatic Analyzer, Hitachi-High-Technologies Corporation, Tokyo, Japan).

Group 3: Renal histological examination.

In another group of animals, we examined the effects of the HSD on renal tissue injury. Seven-week-old DSS rats were treated with the NSD (*n* = 10) or the HSD (*n* = 20) for 10 weeks. Kidney tissues were obtained at 7, 9, 12, 15, and 17 weeks of age (*n* = 2–4 per group/age). Blood was collected from the abdominal aorta with ethylenediaminetetraacetic acid under sodium pentobarbital anesthesia (65 mg/kg, intraperitoneal), and the kidneys were perfused with saline solution. Plasma Cr, blood urea nitrogen (BUN), and sodium were measured by an automated analyzer machine (7020-Automatic Analyzer, Tokyo, Japan). Animals were euthanized with an overdose of pentobarbital (250 mg/kg, intraperitoneal). Renal tissues were dissected and fixed with 10% buffered paraformaldehyde, embedded in paraffin, and sectioned into 3-μm-thick slices. The sections were then stained with periodic acid-Schiff (PAS) or Mallory Azan reagent to evaluate glomerular and tubulointerstitial lesions, respectively. The percentage of the PAS-positive area in each experimental group was measured using image analysis software (WinROOF; Mitani Co., Tokyo, Japan). A total of 30−35 glomeruli were examined for each rat, and the average percentage of the affected lesions was calculated for each rat. The extent of the interstitial fibrotic area was evaluated quantitatively by image analysis software, which determined the area occupied by interstitial tissue positive for Azan staining, as described previously [33]. All morphometric measurements were performed in a blinded manner to avoid any bias.

### 4.2. Statistical Analysis

All data are presented as mean ± standard error of the mean. Statistical comparisons of differences between the groups were performed using one- or two-way analysis of variance combined with the Newman–Keuls post hoc test. Correlation analysis of BP with urinary protein and kidney tissue injury was performed with a simple Pearson regression. For all tests, values of *p* < 0.05 were considered statistically significant.

## Figures and Tables

**Table 1:** Urinary excretion rate of Na, plasma Cr, and BUN concentration in DSS rats.

Age (Weeks)	Urinary Sodium (UnaV), mmol/day		Plasma Cr (mg/dL)		BUN (mg/dL)	
	NSD	HSD	NSD	HSD	NSD	HSD
7	0.25 ± 0.03	Before HSD	0.24 ± 0.02	Before HSD	51 ± 2	Before HSD
		0.30 ± 0.02		0.22 ± 0.02		54 ± 2
9	0.30 ± 0.03	2 weeks after	0.24 ± 0.01	2 weeks after	52 ± 3	2 weeks after
		25.64 ± 0.85 *^†^		0.23 ± 0.01		57 ± 3
12	0.36 ± 0.02	5 weeks after	0.28 ± 0.02	5 weeks after	53 ± 1	5 weeks after
		23.90 ± 0.40 *^†^		0.26 ± 0.04		59 ± 2
15	0.52 ± 0.06 *	8 weeks after	0.30 ± 0.05	8 weeks after	51 ±1	8 weeks after
		25.60 ± 1.15 *^†^		0.24 ± 0.03		63 ± 1
17	0.44 ± 0.05 *	10 weeks after	0.29 ± 0.03	10 weeks after	53 ± 1	10 weeks after
		20.76 ± 0.80 *^†^		0.26 ± 0.04		61 ± 2
21	0.44 ± 0.05 *	4 weeks after switching to NSD	0.30 ± 0.04	4 weeks after switching to NSD	53 ± 1	4 weeks after switching to NSD
		0.76 ± 0.19 *		0.27 ± 0.04		44 ± 5

DSS, Dahl salt-sensitive; NSD, normal salt diet (0.3% NaCl); HSD, high salt diet (8% NaCl); BUN, blood urea nitrogen. * *p* < 0.05 vs. baseline with NSD; ^†^ *p* < 0.05 vs. age matched rats with NSD.

## Supplementary Materials

The following are available online at https://www.mdpi.com/1422-0067/21/6/2248/s1. Figure S1. Body weight in HSD-fed Dahl salt-sensitive rats during the experimental period; Figure S2. Circadian rhythm of systolic blood pressure (SBP) during feeding NSD (at week-7); Figure S3. Circadian rhythm of SBP during feeding HSD (at week-8); Figure S4. Circadian rhythm of SBP during feeding HSD (at week-10); Figure S5. Circadian rhythm of SBP during feeding HSD (at week-17), Figure S6. Circadian rhythm of SBP after switching to normal salt diet (at week-18); Figure S7. Circadian rhythm of SBP after switching to NSD (at week-21); Figure S8. Relationship of SBP and the level of urinary protein excretion; Figure S9. Relationship of SBP and renal histological changes; Figure S10. Urine volume in HSD-fed Dahl salt-sensitive rats.
