# Supplementary material for: Association of a Disrupted Dipping Pattern of Blood Pressure with Progression of Renal Injury during the Development of Salt-Dependent Hypertension in Rats
Source: Int J Mol Sci. 2020 Mar 24;21(6):2248. doi: 10.3390/ijms21062248 (PMC7139748; doi:10.3390/ijms21062248)
Supplement: Supplementary file 1 [file ijms-21-02248-s001.zip › supplementary file/ijms-740053 figures.pdf]

Figure S1

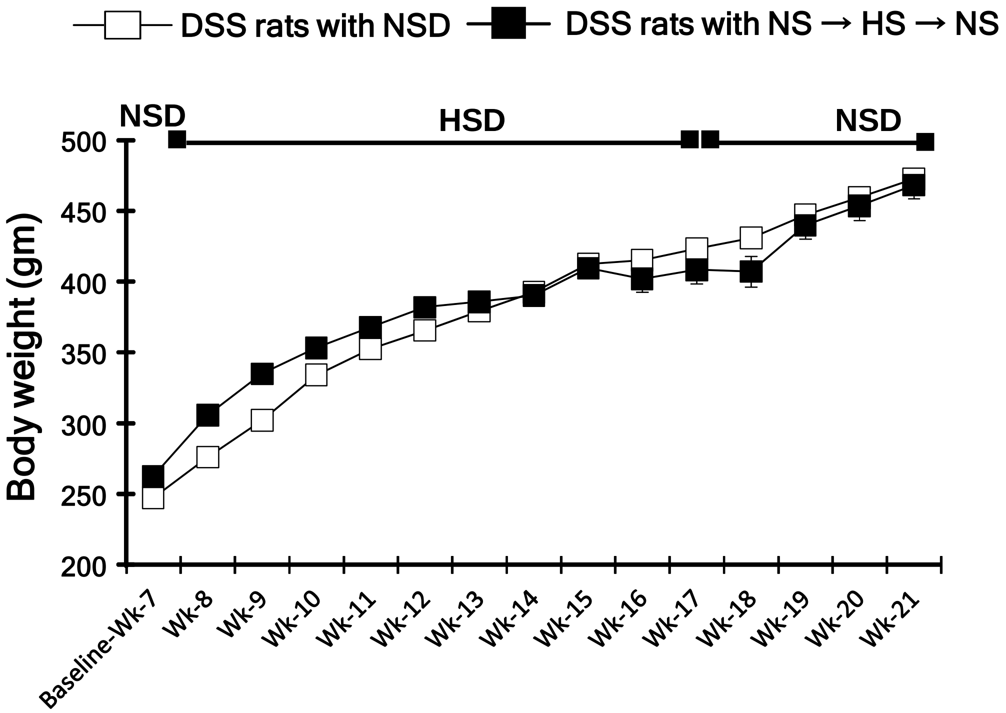
DSS rats with NSD
DSS rats with NS → HS → NS
NSD
HSD
NSD
Body weight (gm)
500
450
400
350
300
250
200
Baseline-Wk-7
Wk-8
Wk-9
Wk-10
Wk-11
Wk-12
Wk-13
Wk-14
Wk-15
Wk-16
Wk-17
Wk-18
Wk-19
Wk-20
Wk-21

Figure S2

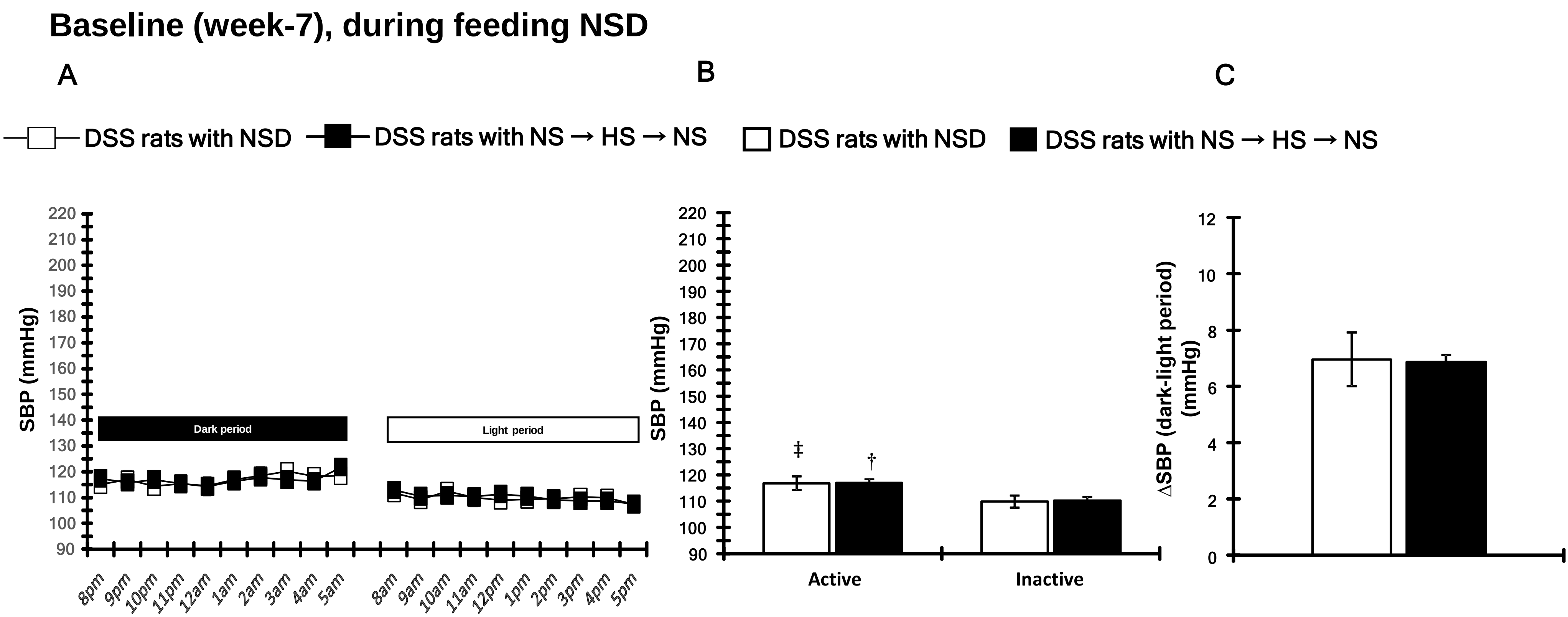

Baseline (week-7), during feeding NSD
A
B
C
DSS rats with NSD
DSS rats with NS → HS → NS
DSS rats with NSD
DSS rats with NS → HS → NS
SBP (mmHg)
Dark period
Light period
8pm
9pm
10pm
11pm
12am
1am
2am
3am
4am
5am
8am
9am
10am
11am
12pm
1pm
2pm
3pm
4pm
5pm
SBP (mmHg)
‡
†
Active
Inactive
ΔSBP (dark-light period) (mmHg)

Figure S3

Five days after HSD (week-8)

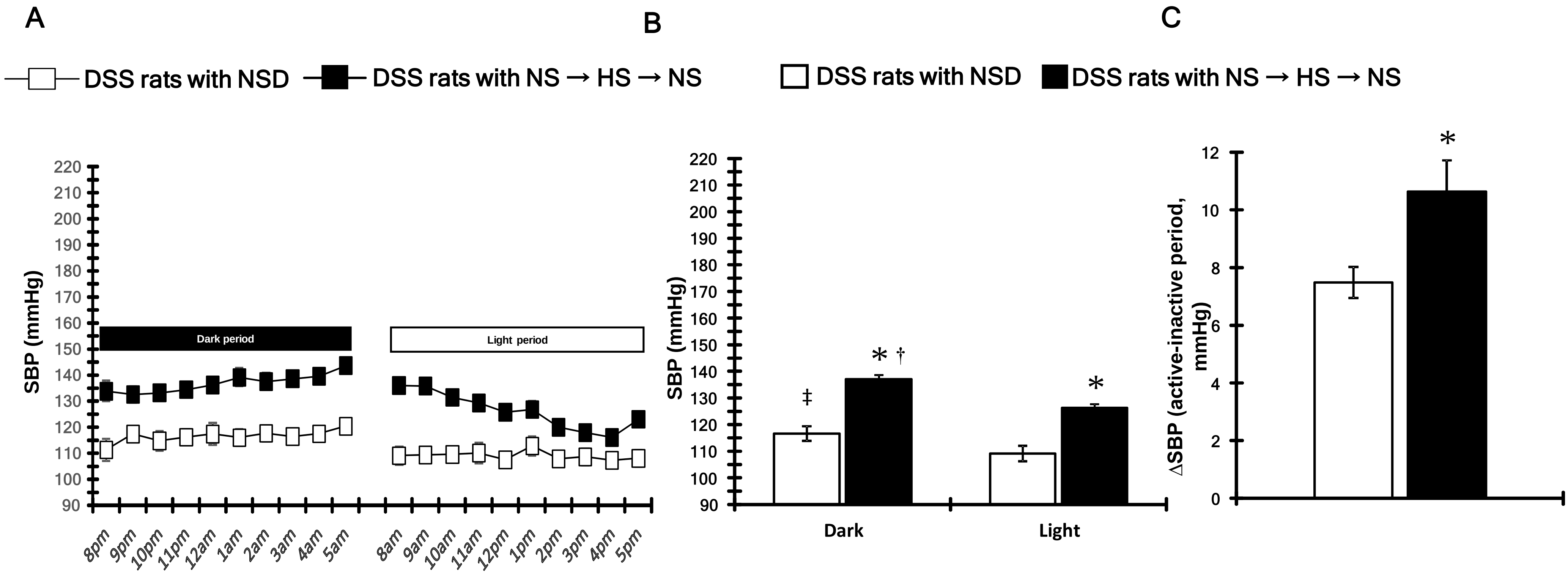

Figure S4

Three weeks after HSD (week-10)

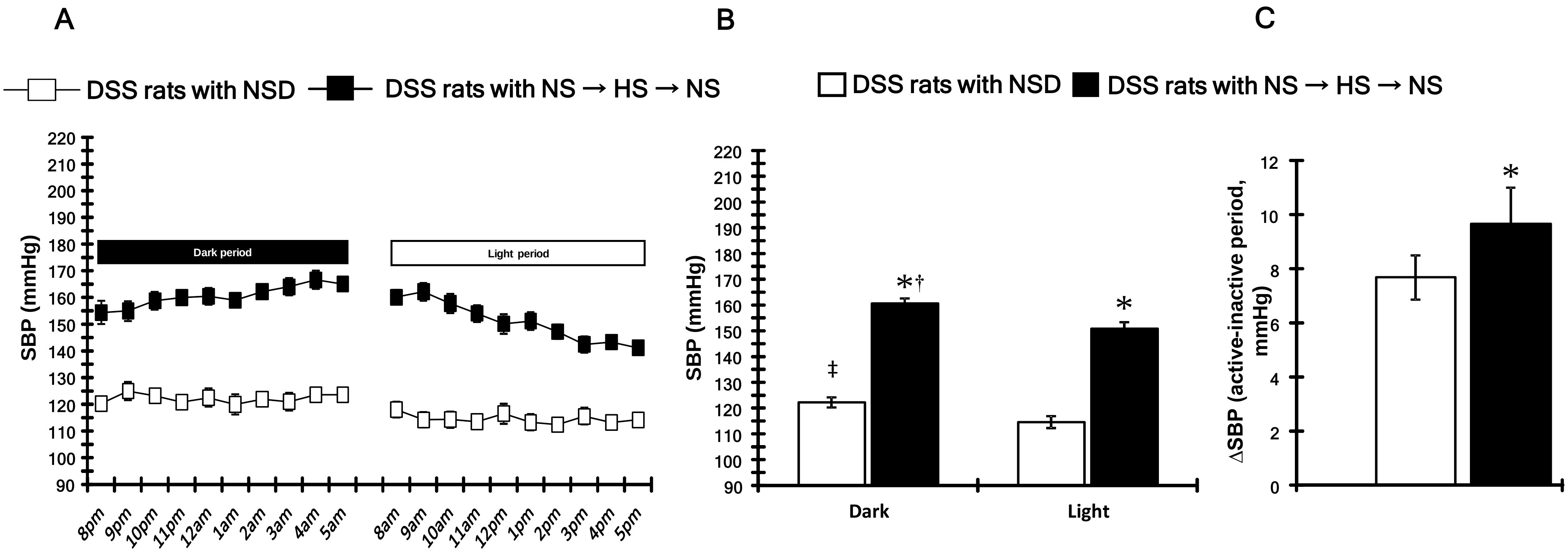

Figure S5

Seven weeks after HSD (week-14)

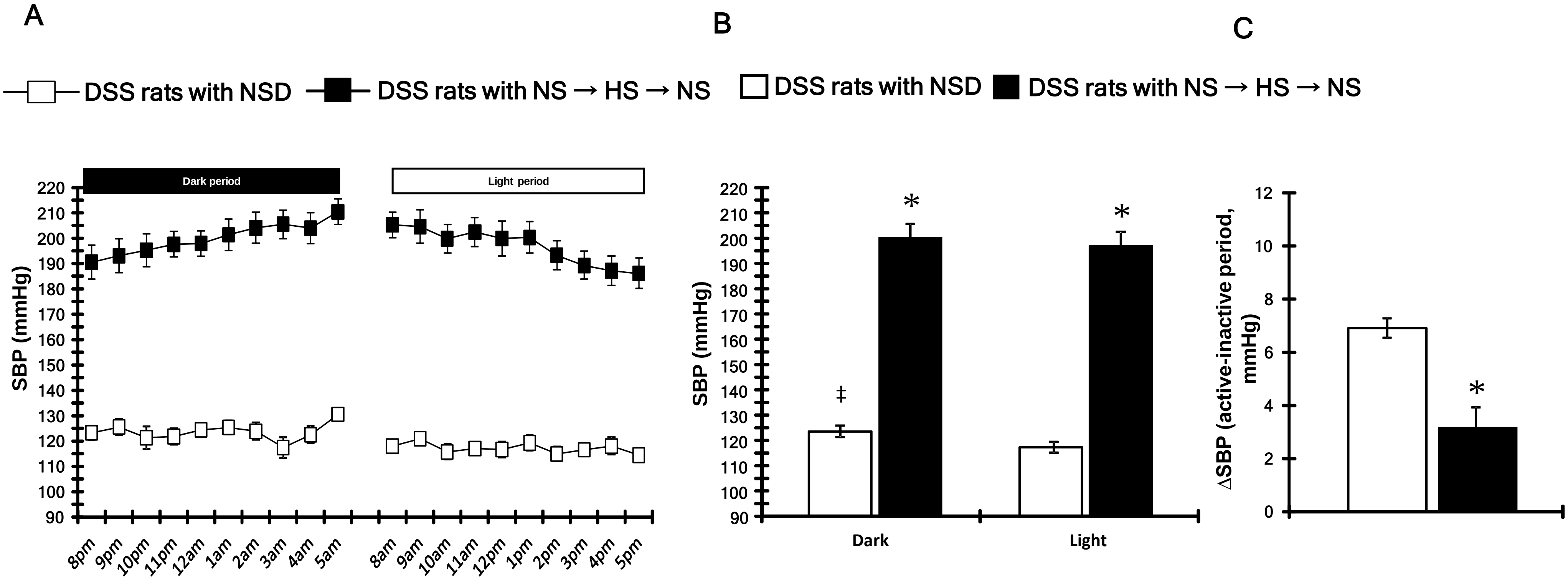

Figure S6

Ten weeks after HSD (week-17)

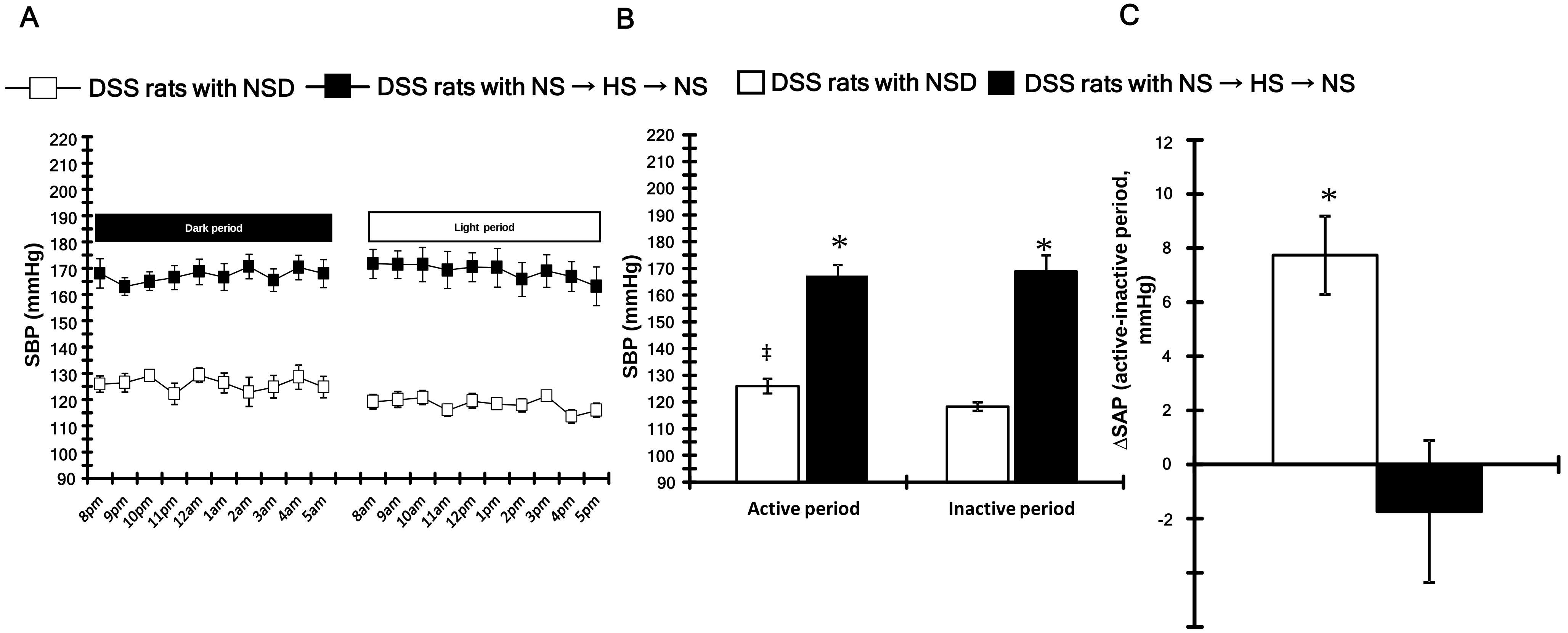

Figure S7

Four weeks after switching HSD to NSD (week-21)

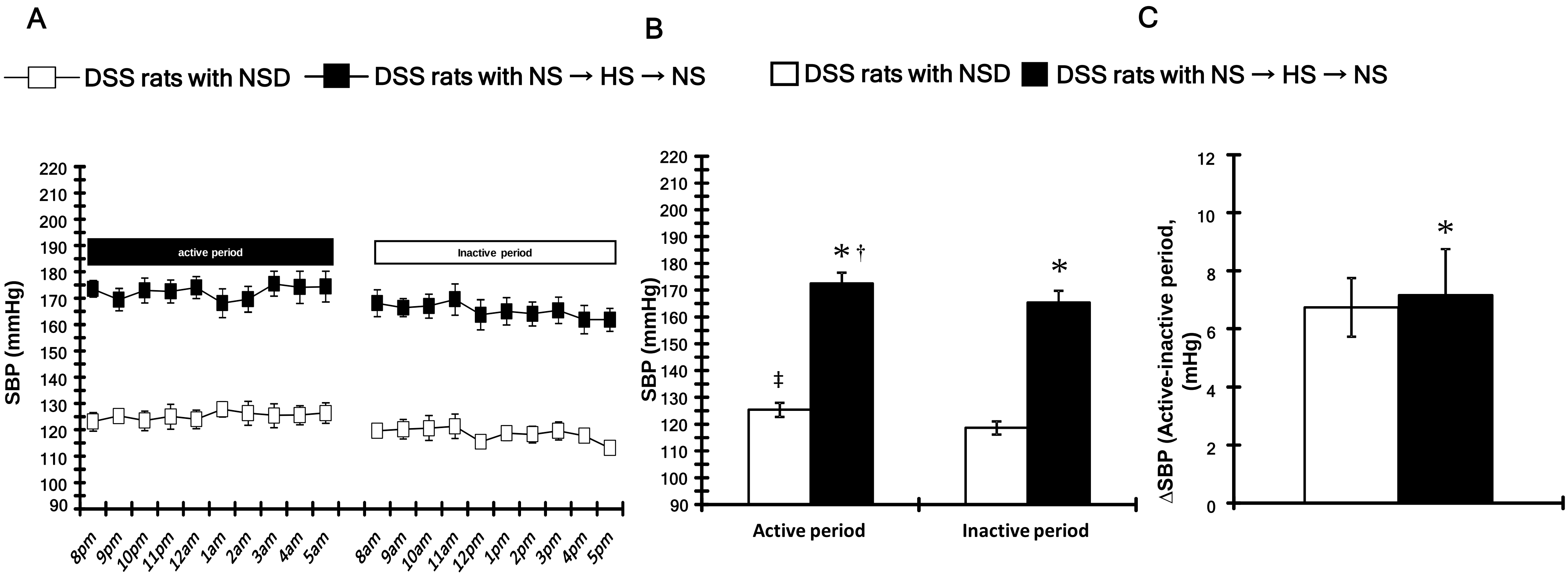

Figure S8

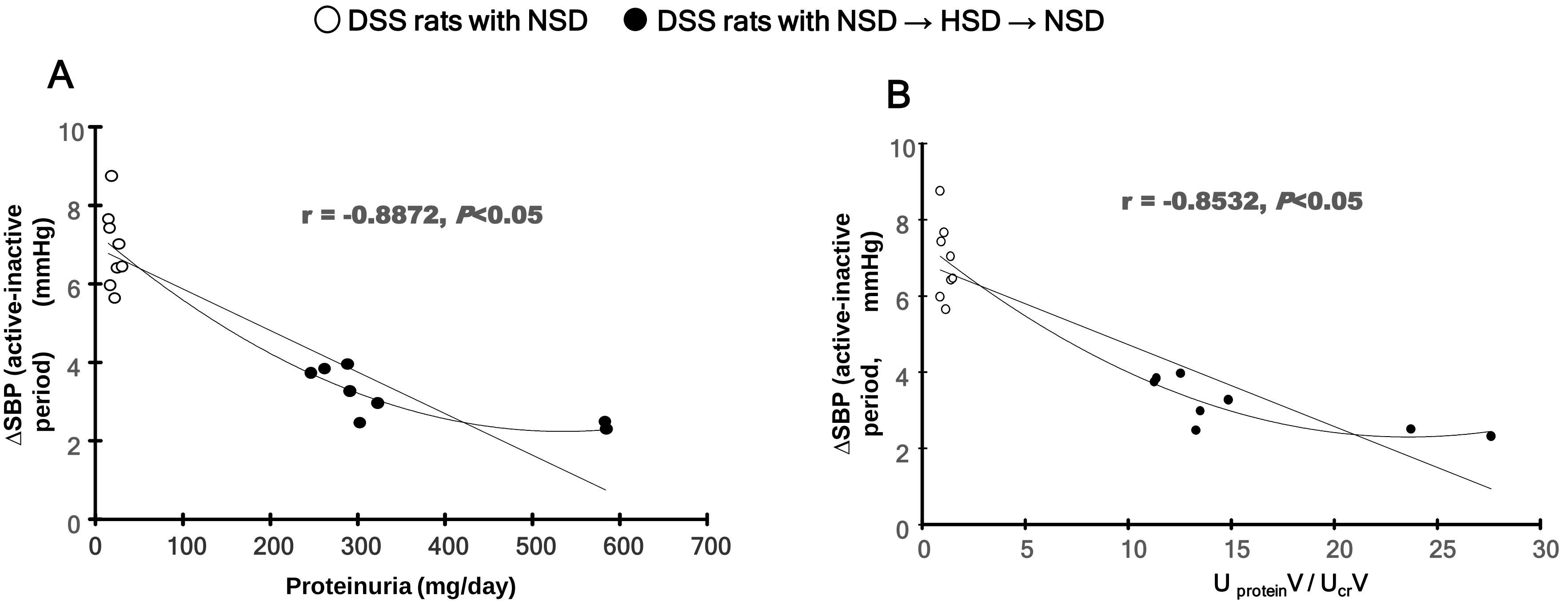

DSS rats with NSD
DSS rats with NSD → HSD → NSD
A
ΔSBP (active-inactive period) (mmHg)
r = -0.8872, P<0.05
0 2 4 6 8 10
0 100 200 300 400 500 600 700
Proteinuria (mg/day)
B
ΔSBP (active-inactive period, mmHg)
r = -0.8532, P<0.05
0 2 4 6 8 10
0 5 10 15 20 25 30
U protein V / U cr V

Figure S9

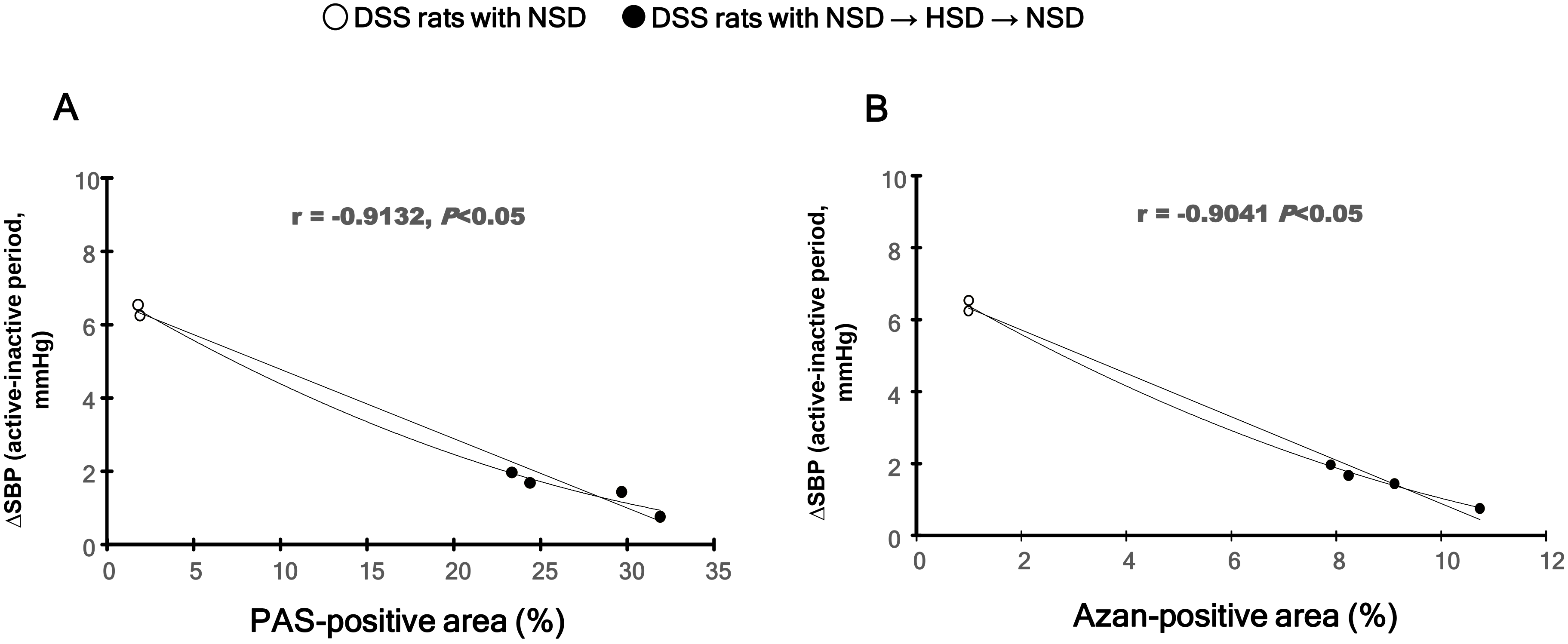

○ DSS rats with NSD
● DSS rats with NSD → HSD → NSD
A
r = -0.9132, P<0.05
ΔSBP (active-inactive period, mmHg)
0
2
4
6
8
10
0
5
10
15
20
25
30
35
PAS-positive area (%)
B
r = -0.9041 P<0.05
ΔSBP (active-inactive period, mmHg)
0
2
4
6
8
10
0
2
4
6
8
10
12
Azan-positive area (%)

Figure S10

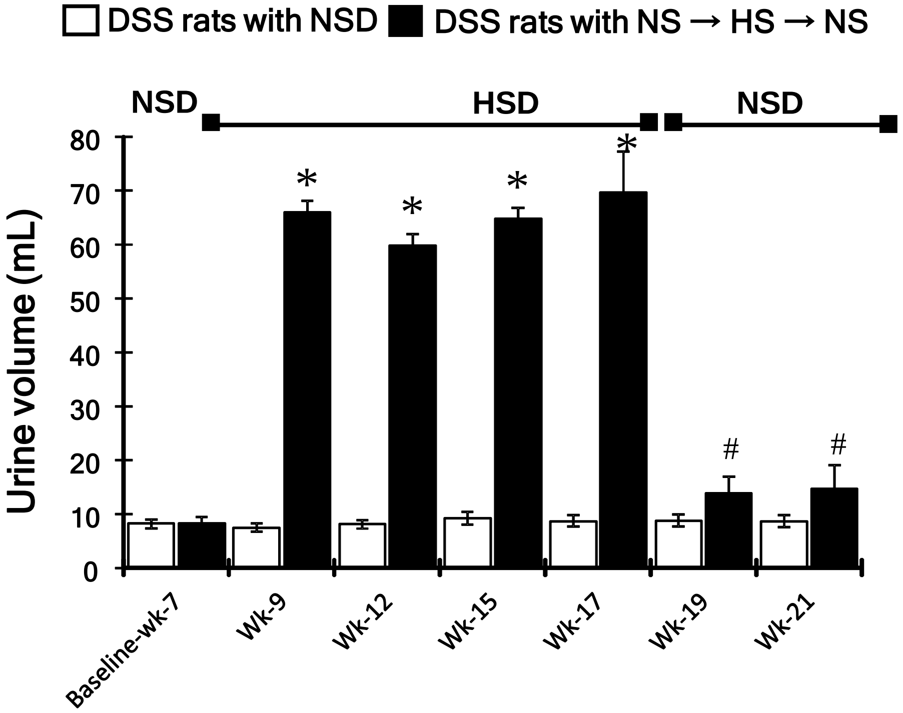
DSS rats with NSD
DSS rats with NS → HS → NS
NSD
HSD
NSD
Urine volume (mL)
80
70
60
50
40
30
20
10
0
*
*
*
*
#
#
Baseline-wk-7
Wk-9
Wk-12
Wk-15
Wk-17
Wk-19
Wk-21
