# Supplementary material for: Association of a Disrupted Dipping Pattern of Blood Pressure with Progression of Renal Injury during the Development of Salt-Dependent Hypertension in Rats
Source: Int J Mol Sci. 2020 Mar 24;21(6):2248. doi: 10.3390/ijms21062248 (PMC7139748; doi:10.3390/ijms21062248)
Supplement: Supplementary file 1 [file ijms-21-02248-s001.zip › supplementary file/ijms-740053 supplementary.pdf]

Supplemental Figure Legends:

Figure S1. Body weight in HSD-fed Dahl salt-sensitive rats during the experimental period.

Figure S2. Circadian rhythm of systolic blood pressure (SBP) during feeding NSD (at week-7). (A) Hourly SBP with NSD, (B) averaged 10-h SBP in active and inactive periods, and (C) the difference of 10-h SBP between active and inactive periods. $^{†}P<0.05$; DSS rats with NSD→HSD→NSD (inactive period) *vs.* DSS rats with NSD→HSD→NSD (active period), $^{‡}P<0.05$; DSS rats with NSD (inactive period) *vs.* DSS rats with NSD (active period).

Figure S3. Circadian rhythm of SBP during feeding HSD (at week-8). (A) Hourly SBP, (B) averaged 10-h SBP in active and inactive periods, and (C) the difference of 10-h SBP between active and inactive periods after 5 days of HSD. *P<0.05 vs. DSS rats NSD; †P<0.05, DSS rats with NSD→HSD→NSD (inactive period) vs. DSS rats with NSD→HSD→NSD (active period); ‡P<0.05, DSS rats with NSD (inactive period) vs. DSS rats with NSD (active period).

Figure S4. Circadian rhythm of SBP during feeding HSD (at week-10). (A) Hourly SBP, (B) averaged 10-h SBP in active and inactive periods, and (C) the difference of 10-h SBP between active and inactive periods after 3 weeks of HSD. *P<0.05 vs. DSS rats with NSD; †P<0.05, DSS rats with NSD→HSD→NSD (inactive period) vs. DSS rats with NSD→HSD→NSD (active period); ‡P<0.05, DSS rats with NSD (inactive period) vs. DSS rats with NSD (active period).

Figure S5. Circadian rhythm of SBP during feeding HSD (at week-17). (A) Hourly SBP, (B) averaged 10-h SBP in active and inactive periods, and (C) the difference of 10-h SBP between active and inactive periods after 10 weeks of HSD. *P<0.05 vs. DSS rats NSD; †P<0.05, DSS rats with NSD→HSD→NSD (inactive period) vs. DSS rats with NSD→HSD→NSD (active period); ‡P<0.05, DSS rats with NSD (inactive period) vs. DSS rats with NSD (active period).

Figure S6. Circadian rhythm of SBP after switching to normal salt diet (at week-18). (A) Hourly SBP, (B) averaged 10-h SBP in active and inactive periods, and

(C) the difference of 10-h SBP between active and inactive periods at 1 week after switching HSD to NSD. *P<0.05 vs. DSS rats NSD; †P<0.05, DSS rats with NSD→HSD→NSD (inactive period) vs. DSS rats with NSD→HSD→NSD (active period); ‡P<0.05, DSS rats with NSD (inactive period) vs. DSS rats with NSD (active period).

Figure S7. Circadian rhythm of SBP after switching to NSD (at week-21). (A) Hourly SBP, (B) averaged 10-h SBP in active and inactive periods, and (C) the difference of 10-h SBP between active and inactive periods at 4 weeks after switching HSD to NSD. *P<0.05 vs. DSS rats NSD;†P<0.05, DSS rats with NSD→HSD→NSD (inactive period) vs. DSS rats with NSD→HSD→NSD (active period); ‡P<0.05, DSS rats with NSD (inactive period) vs. DSS rats with NSD (active period).

Figure S8. Relationship of SBP and the level of urinary protein excretion. (A) Correlation between the difference in active and inactive period SBP (△SBP) and urinary protein excretion. (B) Correlation between the difference in active and inactive period SBP (△SBP) and urinary protein-Cr ratio.

Figure S9. Relationship of SBP and renal histological changes. (A) Correlation between the difference in active and inactive period SBP (△SBP) and glomerulosclerosis. (B) Correlation between the difference in active and inactive period SBP (△SBP) and tubulointerstitial injury.

Figure S10. Urine volume in HSD-fed Dahl salt-sensitive rats. *P<0.05 vs. DSS rats with NSD; #*P*<0.05, DSS rats with NSD→HSD→NSD (week 17) *vs.* DSS rats with NSD→HSD→NSD (week18-21).
